# Coinfection by Hepatitis C Is Strongly Associated with Abnormal CD4/CD8 Ratio in HIV Patients under Stable ART in Salvador, Brazil

**DOI:** 10.1155/2015/174215

**Published:** 2015-08-18

**Authors:** Clara Brites-Alves, Eduardo Martins Netto, Carlos Brites

**Affiliations:** Laboratorio de Pesquisas em Infectologia, Complexo Hospital Universitário Professor Edgard Santos, Universidade Federal da Bahia, Salvador, Brazil

## Abstract

Proper immune restoration (CD4 count >500 and normal CD4/8 ratio) is reached only by a fraction of HIV patients, despite stable viral suppression. *Methods.* We present a case-control study to compare HIV patients with viral suppression >1 year, according to immune restoration pattern: adequate response (AR) defined by CD4 > 500 cells/mm^3^ and CD4/8 ratio >1; partial response (PR = patients with CD4 > 500, but CD4/8 ratio <1); inadequate response (IR = CD4 < 500 cells).* Results.* We evaluated 293 consecutive patients (89 AR, 112 PR, and 92 IR), 70% males. Male gender (*p* < 0.01), lower mean CD4 nadir (*p* < 0.001), higher baseline VL (*p* = 0.01), previous diagnosis of Tb (*p* = 0.03), or HCV (*p* < 0.01) was associated with IR. Likelihood of AR/PR was similar regardless of gender, after adjusting for nadir CD4+ cells count. Longer time under suppressive ART was also associated with a greater chance of AR, but logistic regression identified coinfection by HCV as the main factor associated with abnormal CD4/CD8 ratio. *Conclusion.* Early initiation of ART and longer time since first undetectable PVL were predictors of AR. Previous HCV diagnosis significantly increases the risk of abnormal CD4/CD8 ratio.

## 1. Introduction

The use of highly active antiretroviral therapy (HAART) dramatically changed the natural history of AIDS. The development of new antiretroviral drugs/classes made treating even highly experienced patients possible, with success rates similar to those obtained in therapy of drug-naïve individuals. These advances extended the life expectancy for early treated AIDS patients but also revealed the increased frequency of noninfectious events presented by treated HIV patients [1–4]. One explanation for this phenomenon resides in the facts that even individuals achieving sustained suppression of HIV RNA plasma viremia often fail in restoring CD4 count to a normal range [5–7].

Progressive CD4+ cell depletion is the main landmark of untreated HIV infection. The immunological goal of antiretroviral therapy is to restore the levels of that lymphocytes subpopulation to normal range [6]. However, only a minority of patients starting ART with CD4+ cells count lower than 200 will reach a CD4 count above 500 cells/mm^3^, despite virological suppression [7–9]. In addition, even individuals presenting with CD4 count above that threshold often fail in achieving a normal CD4/CD8 ratio (equal to or above 1.0). Failure to reach a normal CD4/8 ratio is associated with an increased frequency of non-AIDS events and death [10, 11].

Several factors have been associated with inadequate immune restoration (failure in reaching a CD4 count >500 cells/mm^3^), such as low CD4 nadir, older age, high pretherapy HIV viral load, and gender [5–9]. However, we have scarce studies focusing on predictors of normalization of CD4/8 ratio. In the present work, we used a case-control design to define the factors driving an adequate CD4 gain and normal CD4/CD8 ratio in HIV patients under suppressive therapy, in Salvador, Brazil.

## 2. Methods

### 2.1. Study Design

We used a 1 : 2, nonmatched, case-control design to investigate the factors associated with different degree of immune restoration: we defined cases (AR, “adequate immune response”) as individuals presenting CD4 > 500 cells/mm^3^ and a CD4/CD8 ratio >1.0; controls were defined as subjects presenting either CD4 >500 cells/mm^3^, but CD4/CD8 ratio <1.0 (PR, “partial immune restoration”), or patients failing to reach CD4 cells count >500 cells/mm^3^ (IR, “inadequate immune restoration”).

### 2.2. Study Population

We selected consecutive HIV patients under stable antiretroviral therapy (ART), and HIV-1 RNA plasma viral load (VL) <50 copies/mm^3^, for, at least, 1 year. All patients were regularly followed at Universidade Federal da Bahia Hospital's (C-HUPES) AIDS clinics, in Salvador, Brazil. C-HUPES is one of the first referral centers for HIV care in Bahia state and attends to about 2,000 patients. We collected data on demographics, present/past coinfections, and current antiretroviral regimen. All previous VL measurements and CD4/CD8 counts were recorded for comparison between groups. For analysis purposes, in case of multiple CD4/CD8 measurements in one year period, we used the highest recorded value.

### 2.3. Sample Size Calculation

We estimated that a minimum of 30% of cases and 50% of controls had CD4 nadir below 200 cells/mm^3^. To achieve 80% power to detect differences between groups (alpha = 0.05) and 95% confidence interval, we needed a minimum of 190 patients (95 cases and 95 controls). To better study the differences between partial immune restoration and failure of immune restoration we included 192 controls.

The study was approved by the institutional IRB (UFBA's School of Medicine, number 055/11).

### 2.4. Statistical Analysis

Patients were included into one of the three study groups: cases (patients with more than one year of controlled viral load and adequate immune restoration, AR) and control groups (individuals with inadequate/partial immune restoration, IR/PR, despite at least one year of controlled viral load). The mean and 95% confidence intervals were calculated for continuous variables (age, nadir CD4+ T-cell counts per cubic millimeter before ART, days after diagnosis until ART initiation, days under ART to achieve viral suppression, and HIV-1 plasma RNA level logarithm before HAART) and compared according to the three immunological outcomes. Proportions among the groups were calculated for gender, marital status, infection route, history of AIDS-defining condition, HTLV, chronic hepatitis C (PCR positive) or hepatitis B (AgHBs positive) infection, and previous tuberculosis (any site) and compared using ANOVA. For analysis' purpose patients were stratified by CD4 nadir, gender, and past diagnosis of tuberculosis or viral hepatitis. And, finally, CD4 nadir was stratified into 7 intervals (0–49; 50–99; 100–199; 200–299; 300–399; 400–499; and 500 or more CD4+ T-cell counts per cubic millimeter) to compare outcomes across groups, using Chi-square statistics. Difference of means and proportions were considered statistically significant if the probability of the event was inferior to 0.05 after adjustment for stratification factors.

Variables significantly associated with immune restoration pattern in bivariate analysis were included in logistic regression model. Absence of adequate immune restoration (control groups) was used as a dependent variable, compared with cases (normal CD4/CD8 ratio and CD4 count > 500 cells/mm^3^). Independent variables included male gender, mean duration of viral suppression, age at diagnosis, and previous diagnosis of HCV or tuberculosis.

Statistical analysis was performed using Statistical Package for Social Sciences (SPSS) version 21.

## 3. Results

A total of 293 patients were consecutively enrolled in the study (92 cases and 189 controls) from June 2013 to June 2014. All patients attending one of the 3 C-HUPES AIDS clinics in this period of time who met the entry criteria were eligible to enter the study.

**Figure 1:**
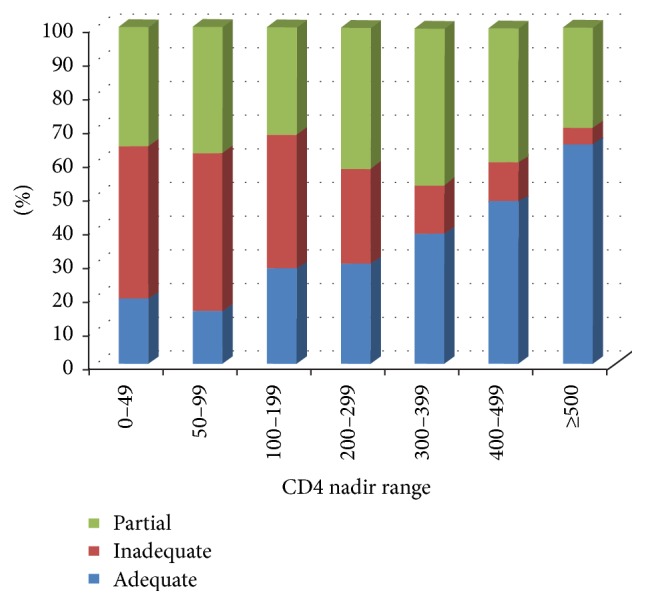
Proportion of patients reaching inadequate, partial, or adequate immune restoration, according to mean CD4 count nadir.

Mean follow-up duration was 1804 ± 1189 days.  Table 1 displays the main demographic characteristics of groups. We detected a higher proportion of MSM patients presenting with inadequate immune restoration in comparison with subjects infected by heterosexual contact, but it did not reach statistical significance. In addition, women were more likely to achieve AR than men, but they had mean CD4 count nadir significantly higher than that observed for men (264.7 ± 176.6 cells/mm^3^ versus 209.8 ± 190.5, resp., *p* < 0.001).  Figure 1 displays the distribution of groups across different CD4 strata.

Lower mean CD4+ cells count nadir and shorter time under suppressive therapy were significantly associated with IR or PR, in comparison with patients presenting AR (Table 1). Patients presenting IR had a mean shorter time (1451 ± 1111 days) since the first undetectable VL, compared to those achieving PR (1680 ± 1076) or AR (2306 ± 1242, *p* = 0.03 for comparison between AR and the other groups).

On the other hand, higher VL at the moment of starting therapy was strongly predictive of IR, but we did not detect difference when comparing mean VL values between PR and AR groups (Table 2). There was also no difference between groups regarding the use of specific ARV regimens (data not shown). Mean time for initiation of ART after diagnosis of HIV was 510 ± 889 days (no difference between groups).

We also detected a significant association between previous diagnosis of either HCV or tuberculosis infection and failure in reaching an adequate immune restoration. Patients coinfected by HCV had a significantly higher likelihood of presenting PR than those without evidence of HCV infection. In addition, past history of tuberculosis was predictive of IR (*p* = 0.03). History of other AIDS-defining infections or HTLV coinfection was not related to the pattern of immune restoration. Tables 2 and 3 summarize clinical and laboratory characteristics of patients according to immune restoration pattern.

**Figure 2:**
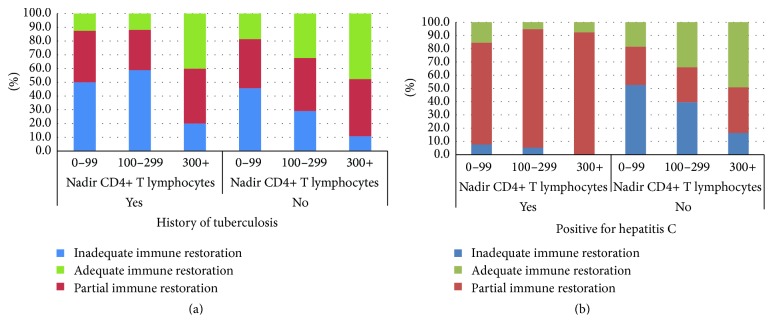
Distribution of patients by immune restoration patterns according to mean nadir CD4 cells and past diagnosis of tuberculosis (a) or HCV infection (b).

The effect of HCV infection on CD4/CD8 ratio is clearly observed in the PR group: most of HIV-HCV coinfected patients presented with CD4 count higher than 500 cells/mm^3^ but a CD4/CD8 ratio below 1.0.  Figure 2 shows the pattern of immune restoration for patients according to past tuberculosis/HCV coinfection and CD4 nadir range.

The likelihood of a patient to achieve a normal (>1.0) CD4/CD8 ratio was lower than 20% for those starting ART with a CD4 count below 100 cells/mm^3^, but it was as high as 65% for those who initiated therapy with CD4 > 500 cells/mm^3^, as shown in Figure 1. We found that around 40% of patients fit the definition of PR, regardless of CD4 nadir. Moreover, among patients who started ART with CD4 count higher than 400 cells/mm^3^ the proportion of IR was only 12%, and it decreases to only 5% for those starting therapy with a CD4 count higher than 500 cells/mm^3^.

CD4 nadir, time under viral suppression, gender, tuberculosis, and hepatitis C coinfection were included in the multivariate analysis model. As shown in Table 4, the associations between previous tuberculosis and mean elapsed time under suppressive therapy disappeared. Only CD4 count nadir and HCV coinfection remained significantly associated with inadequate/partial immune restoration pattern.

## 4. Discussion

Our results demonstrate the degree of immune restoration after suppressive ART is defined by multiple factors, including the moment of ART initiation and duration of suppression of HIV-1 plasma viremia. In addition, past tuberculosis or HCV coinfection negatively impact the magnitude of immune restoration of patients. Regarding tuberculosis, since the majority of cases occurred in patients with CD4 count < 200 cells/mm^3^, it appears to be a marker of low CD4+ cells count nadir, rather than an independent factor for inadequate immune restoration. On the other hand, CD4 count nadir and HCV infection were clearly associated with failure to achieve a CD4/CD8 ratio above 1.0.

Persistent immune activation (PIA) is a phenomenon associated with the development of non-AIDS events in patients under suppressive ART [12–14]. A marker of PIA is a CD4/CD8 ratio below 1.0 and the magnitude of decrease in CD4/CD8 ratio is directly associated with risk of non-AIDS events or death [10, 11]. Mussini et al. demonstrated that a CD4/CD8 ratio below 0.8 was significantly associated with a higher risk of noninfectious complications of AIDS, and the risk of death increased with a ratio below 0.4 [15]. In the present work, most of HCV coinfected patients presented with a CD4/CD8 ratio below 1.0, regardless of CD4 nadir, confirming HCV as an important factor impairing immune response.

Several conditions have been implicated as cause of persistent immune activation: bacterial translocation, chronic viral coinfections, and low-level HIV replication are potential causes of PIA [12, 13]. Among viral infections, CMV is considered a potent immune activator, and even in patients without immunodeficiency it can activate both CD4+ and CD8+ lymphocytes [16]. In addition, coinfection by HCV is recognized as a potential cause of deficient increase in CD4+ cells, after suppressive ART. In a recent study, Taye et al. showed that HCV coinfection was associated with poorer recovery in CD4+ cells, in comparison with HCV negative patients [17]. However, other authors did detect neither a negative impact of HCV on immune restoration nor a linkage between the degree of immune impairment and duration of suppressive ART [17–20].

A meta-analysis of 8 trials, involving 6,206 patients concluded that coinfection by HCV is an independent factor associated with poorer CD4 recovery, in comparison with HIV infection alone [21]. Our findings demonstrate that HCV coinfection does not impair CD4+ absolute cells count recovery but it reduces the chance of patients to achieve a normal CD4/CD8 ratio. This fact reinforces the role of HCV as a predictive factor in PIA.

Our results are also in accordance with the available evidence about the effect of a low nadir CD4 count on the likelihood of reaching an adequate immune restoration. Recently published studies concluded that this variable is an important predictor of immune response following successful ART [22, 23]. Our findings also confirm that time might matter in terms of immune restoration: patients achieving AR had significantly longer time since the first undetectable HIV-1 PVL in comparison with those who failed in achieving a CD4/CD8 ratio >1.0. However, there was no difference when we compared PR and IR groups, and the significance of such finding disappeared in multivariate analysis. Since a strong predictor of CD4+ recovery in our study was CD4+ cells nadir, it indicates that normalization of CD4/CD8 ratio is also time dependent and can rely on other patient's characteristics.

Interestingly, the PR group presented the higher CD4+ gain per year after achieving virological suppression. PR group had a mean net gain of 192 cells/mm^3^, while in CR patients it was equal to 156 cells/mm^3^. Patients starting ART with higher CD4+ counts will need less increase of these cells to reach a normal range. On the other hand, patients in lower CD4+ strata would need a higher CD4+ cells gain, to reach normal levels, and probably had a more severe impairment in their capacity to reach a proper immune restoration. This finding was also observed in a previous study on HIV-HCV coinfection [18].

We did not find an effect of age on CD4 recovery or CD4/CD8 ratio normalization. In a recent, large study conducted in Zambia, older age was significantly associated with a poor CD4 outcome [24]. However, the mean baseline CD4 count was much lower than that observed in our work, with most patients starting ART presenting with a CD4 count below 150 cells/mm^3^. It reinforces the evidence that CD4 nadir would increase the risk of inadequate immune recovery, especially for elderly patients.

CD4/CD8 ratio is considered a marker of immunosenescence, but its role as a predictor of non-AIDS event is still controversial [10–12]. In a recent work, Saracino showed that it does not seem to be related to chronic inflammation, but it is a clear sign of immune dysregulation that could be associated with metabolic complications [25].

Assuming that normalization of CD4/CD8 ratio decreases the risk of non-AIDS events, our findings indicate that the current “test and treat” strategy adopted by some countries (including Brazil) to fight AIDS probably will reduce the proportion of treated patients failing to achieve an adequate immune restoration, once the early treatment will increase their chances to meet that goal. This can be an important intervention to reduce the burden of non-AIDS events that characterizes the current face of AIDS epidemic in developed countries, since the available evidence suggests that they are associated with inadequate immune restoration after suppressive ART.

Our study has some limitations. We included patients diagnosed in the pre-HAART era, which could introduce some bias in our results. However, it is important to know how patients with long-term follow up behave in terms of immune recovery. This subgroup provides us with the opportunity to evaluate the pattern of immune restoration for over 10 years of treatment and to define whether immune restoration is time dependent or whether it is affected by ART specific regimens. In addition, the use of two control groups made it possible to evaluate differences in the pattern of immune restoration in this population.

An open question remains on HCV impact: there is no data on the effects of HCV treatment on immune recovery in coinfected patients. Will HCV eradication suppress the negative impact of this coinfection on immune restoration or will it persist even after successful treatment? Larger, prospective, well-conducted studies, focused on the impact of HCV clearance on immune response of coinfected patients, are needed to provide a definitive answer to this question.

## Figures and Tables

**Table 1:** Characteristics of HIV patients under stable, suppressive antiretroviral therapy, according to pattern of immune restoration.

Patients characteristics	Immune restoration group			*p* value
	Inadequate immune restoration	Partial immune restoration	Adequate immune restoration	
	(*N* = 89)	(*N* = 112)	(*N* = 92)	
Age at HIV diagnosis–yr				
Mean (95% CI)	46.2 (44.3–48.0)	48.5 (46.7–50.4)	48.4 (46.4–50.4)	NS
Male sex, number (%)	62 (69.7)	74 (66.1)	44 (47.8)	<0.01
Living without spouse or partner, number (%)	59 (66.3)	73 (65.2)	50 (54.3)	NS
Route of infection, number (%)				
Heterosexual	40 (44.9)	63 (56.3)	56 (60.9)	NS
Homo-/bisexual	45 (50.6)	46 (41.1)	35 (38.0)	
Other	4 (4.5)	3 (2.7)	1 (1.1)	

NS: not significant.

**Table 2:** Clinical and laboratory characteristics of patients according to degree of immune restoration.

Pattern of immune restoration				
Previous infections	Inadequate	Partial	Adequate	*p* value
	*N* (%)	*N* (%)	*N* (%)	
History of AIDS defining infection	25 (28.1)	45 (40.2)	26 (28.3)	NS
Hepatitis C^*∗*^	2 (2.2)	43 (38.4)	4 (4.3)	<0.01
Hepatitis B	1 (1.1)	2 (1.8)	3 (3.3)	NS
HTLV	1 (1.1)	4 (3.6)	0 (0)	NS
Tuberculosis	16 (18.0)	12 (10.7)	7 (7.6)	0.03

^*∗*^For comparison, between AR group and PR or IR groups. No difference was found for comparison between IR and PR groups.

NS: not significant.

**Table 3:** Mean HIV-1 RNA plasma viral load and and CD4 count of patients at baseline, according to immune restoration pattern.

Pattern of immune restoration							
Variables	Inadequate		Partial		Adequate		*p* value
	*N*	Mean (95% CI)	*N*	Mean (95% CI)	*N*	Mean (95% CI)	
CD4 nadir							
All patients	89	164 (119–173)	112	230 (199–260)	92	314 (268–360)	<0.001
Males	62	121.7 (105.6)		208.9 (164.4)		336.1 (248.5)	<0.001
Females	27	204.0 (157.0)	38	270.5 (155.0)	48	294.3 (196.7)	0.1
Pretherapy plasma viral load (log_10_)^*∗*^	77	5.0 (4.8–5.1)	87	4.6 (4.5–4.8)	73	4.7 (4.5–4.9)	0.01
Mean duration (days) of viral suppression^*∗*^	74	1451 ± 1111	90	1680 ± 1076	74	2306 ± 1242	0.03^*∗*^
Mean elapsed time (days) between HIV diagnosis and ART initiation (95% CI)^*∗*^	77	383 (229–537)	112	636 (436–836)	92	519 (314–725)	NS

^*∗*^12 patients started ART before viral load tests were available in C-HUPES (missing values).

**Table 4:** Multivariate analysis of factors associated with different patterns of immune restoration.

Inadequate (plus partial) versus adequate immune restoration^*∗*^			
Variable	Odds Ratio	95% CI	
Lower mean CD4 nadir	1,002	1,001	1,004
Duration of virological suppression	1,000	1,000	1,000
Male gender	1,983	,971	4,048
Hepatitis C	18,748	5,309	66,210
Tuberculosis	2,046	,630	6,640

^*∗*^Adequate immune restoration: CD4 > 500 cells/mm^3^, and CD4/CD8 ratio > 1.0; Partial immune restoration: CD4 count > 500 cells/mm^3^, but CD4/CD8 ratio < 1.0; inadequate immune restoration: CD4 < 500 cells/mm^3^ and CD4/CD8 ratio < 1.0.

## Summary

In Brazilian HIV patients under suppressive ART the pattern of immune restoration was related to CD4 nadir, higher baseline plasma HIV RNA viral load, and time under suppressive ART. Past HCV infection was associated with incomplete immune restoration.
